# Implementation of brief dialectical behavior therapy skills training among borderline personality disorder patients in Malaysia: feasibility, acceptability, and preliminary outcomes

**DOI:** 10.1186/s12888-021-03500-y

**Published:** 2021-10-04

**Authors:** Shian-Ling Keng, Hajar Binti Mohd Salleh Sahimi, Lai Fong Chan, Luke Woon, Choon Leng Eu, Su Hua Sim, Man Kuan Wong

**Affiliations:** 1 Division of Social Science, Yale-NUS College, Singapore, Singapore; 2 Department of Psychiatry, Hospital Canselor Tuanku Muhriz, National University of Malaysia (UKM), Cheras Kuala Lumpur, Malaysia; 3 Department of Psychology, HELP University, Bukit Damansara Kuala Lumpur, Malaysia

**Keywords:** Dialectical behavior therapy, Borderline personality disorder, Cross cultural research, Self-harm behaviors

## Abstract

**Aim/background:**

Even though dialectical behavior therapy (DBT) has received substantial empirical support in treating patients with borderline personality disorder (BPD), fewer studies have evaluated whether a brief DBT skills group may be effective in improving clinical outcomes in this population. Further, less is known regarding the feasibility and outcomes of DBT beyond Euro-American contexts. This paper describes outcomes from a pilot study examining the feasibility, acceptability, and clinical outcomes following completion of a shortened, 14-week DBT skills group in a sample of Muslim-majority BPD patients in Malaysia.

**Methods:**

Twenty patients were recruited from a public hospital and attended DBT skills groups in an outpatient clinic. Participants completed measures assessing psychological symptoms, self-harm behaviors, suicidal ideation, emotion regulation difficulties, self-compassion, and well-being pre- and post-intervention.

**Results:**

There were significant reductions in depressive symptoms, stress, and emotion regulation difficulties, as well as increases in self-compassion and well-being from pre- to post-intervention. A trend was found for decreases in frequency and types of non-suicidal self-harm behaviors, suicidal ideation, and anxiety symptoms. Qualitative content analyses of participants’ feedback indicated that the vast majority of participants perceived a positive impact from the skills group, with mindfulness and distress tolerance being rated frequently as skills that were beneficial.

**Conclusion:**

These preliminary findings suggest that DBT skills training is feasible and acceptable in a Muslim-majority, low resource clinical setting, and holds promise in improving clinical outcomes among BPD patients in Malaysia.

**Supplementary Information:**

The online version contains supplementary material available at 10.1186/s12888-021-03500-y.

Borderline personality disorder (BPD) is a severe mental health condition characterized by dysregulated affect, behaviors, cognition, and interpersonal relationships [1]. It is a challenging to treat condition given its association with an increased risk for suicide as well as a high rate of self-harm behaviors [2, 3]. BPD is also highly co-morbid with other psychological disorders, such as major depression and anxiety disorders [4], and is associated with frequent utilization of healthcare services and high costs to the society [5]. Among various treatment approaches, dialectical behavior therapy (DBT) has demonstrated efficacy in improving clinical outcomes among BPD patients [6]. Given that the majority of studies evaluating the effects of DBT were conducted in North American or European contexts, less is known regarding the extent to which the efficacy of DBT (and its components) generalizes cross culturally. Further, little work has explored the potential effects of brief DBT skills training for patients diagnosed with BPD. In this study, we aimed to evaluate the feasibility and outcomes of a 14-week DBT skills group in the Malaysian context.

To date, there is a scarcity of research examining the prevalence of BPD and other personality disorders in Malaysia. Located in Southeast Asia, Malaysia is a multicultural country (formerly a British colony) made up of 32.7 million people, with approximately 70% being Malay Muslims, and 22.6 and 6.8% being Chinese and Indians respectively [7]. In a study involving convenience sampling [8], 14% of patients hospitalized for deliberate self-harm in a tertiary general hospital in Malaysia were found to meet criteria for BPD. In another study, BPD was identified as common psychiatric comorbidity (2.9%) among female inmates in a drug rehabilitation center [9].

Within clinical settings in Malaysia, BPD is still largely under-diagnosed, under-treated and stigmatized, even though anecdotal observations indicated a consistent rise of individuals seeking treatment for BPD-related presentations in public hospitals. Often, psychiatric hospitalization is the first treatment option for high risk patients presenting with self-harm or suicidal behaviours. This poses a major challenge simultaneously as Malaysian psychiatric wards are generally understaffed, with nurses with a lack of training to handle the complex behaviors of patients with BPD. From DBT’s perspective, hospitalizations and use of crisis services may reinforce self-harm and suicidal behaviors iatrogenically [10, 11], though there has not been systematic research tracking longitudinal outcomes following hospitalizations among BPD patients in Malaysia.

While some mental health clinicians are more open to providing a BPD diagnosis relative to others, many struggle with providing sustained and effective treatment for these patients. There is therefore a critical need for Malaysian clinicians be trained to provide evidence-based treatments for patients with BPD, and for research to evaluate the feasibility and effects of these treatments within the local cultural context.

## Efficacy of DBT skills training

Among several evidence-based approaches, DBT has received empirical support in its efficacy in treating patients with BPD and other conditions, such as binge eating disorder and treatment-resistant depression [6, 12, 13]. As a multimodal treatment package, standard DBT consists of four components, namely individual sessions, DBT skills group, phone coaching, and consultation team [14]. Despite DBT’s established efficacy in treating symptoms of BPD and other disorders, implementation of the full treatment package takes up significant resources, due to the multi-component nature of the treatment. In low resource settings where manpower, training, and financial resources are limited, implementation of a full, standard DBT protocol may not be feasible. Therefore, there is emerging research evaluating various adaptations of DBT, including delivery of components of the treatment in a shorter duration. Of the four modes of DBT, the skills group has received the most research attention, given the relative ease of transferability of the skills, and also the fact that DBT skills have been found to be key processes underlying clinical outcomes resulting from DBT [15, 16]. For example, improvements in mindfulness and distress tolerance skills have been found to predict improvements in general psychopathological symptoms resulting from DBT skills training [16].

Several studies have examined the effects of DBT skills training alone on BPD patients [17]. A naturalistic study found that patients completing one DBT skills group cycle (lasting 24 weeks) showed significant improvements in BPD symptoms, depression, and suicidal ideation [18]. In another study, a 13-week DBT skills training program was found to be more effective than standard group therapy (led by psychodynamic-oriented therapists) in reducing dropout rates and improving depressive and anxiety symptoms, anger, irritability, and affect instability among BPD patients [19]. No between-group differences on self-harm or suicidal behaviors were observed in this study. Another study found that one-year of DBT skills training was more effective in reducing self-harm behaviors and depressive symptoms among suicidal BPD patients compared to DBT individual therapy alone [20]. Both treatment conditions were equally effective in reducing suicidal ideation and attempts. Compared to those receiving skills training alone, patients receiving individual therapy in conjunction with skills training were less likely to drop out [20]. In line with findings from this study, a study demonstrated that a 20-week DBT skills group resulted in significant reductions in suicidal and self-harm behaviors and anger, as well as improvements in distress tolerance and emotion regulation compared to a waitlist control condition [21].

While the findings suggest that DBT skills training alone is effective in improving selected clinical outcomes among BPD patients, fewer studies have examined the extent to which briefer forms of DBT skills training (e.g., less than 20 weeks in duration) may bring about clinical benefits [19]. In one study, a 16-week DBT skills group was found to be effective in reducing emotional dysregulation and anxiety in a sample of individuals with mixed depressive or/or anxiety disorders [22]. Two studies focusing on college students with BPD or cluster B personality traits found evidence for the effects of shortened (i.e., between eight and eleven weeks) DBT skills training in reducing depression and BPD traits [23], and in increasing skills use and decreasing maladaptive coping [24]. Despite the promising findings, it is unclear whether these benefits would generalize for BPD patients in a clinical setting.

Beyond a focus on psychopathological symptoms, the field could benefit from examining well-being related outcomes resulting from skills training. For example, given the emphasis on mindfulness (and associated skills such as letting go of judgments of oneself) as a core skillset in DBT, participation in a DBT skills group may be associated with improvements in well-being and self-compassion, outcomes that have been reported across multiple studies examining the effects of mindfulness-based interventions [25–27].

## Implementation of DBT skills training in diverse cultural and low resource contexts

Further, as most studies on DBT skills training published to date were conducted in North American or European contexts, little is known regarding the extent to which the effects of DBT skills training (or for that matter, DBT as a comprehensive treatment package) would generalize across cultural contexts, including contexts with low trained manpower and mental health resources. Within Asia, DBT skills training has been adapted for suicidal women in rural Nepal, with preliminary evidence supporting its effects in improving emotion regulation and reducing suicidal ideation [28]. In Taiwan, DBT has been adapted for Chinese-speaking patients with BPD, and results from an open-label trial showed that participation in DBT was associated with reductions in suicidal behaviors and ideations, depressive symptoms, and BPD symptoms [29].

Several aspects of DBT, such as its reliance on principles (rather than fixed or rigid protocols), flexibility, and collaborative nature make the treatment easily adaptable for different groups and populations [30]. For example, as opposed to imposing a set of pre-established goals and values, the treatment involves a careful assessment and identification of clients’ individual, behavioral goals, along with consideration of ways in which DBT skills can be applied towards achieving these goals. While several DBT skills such as mindfulness and radical acceptance are drawn from Zen philosophy and Buddhism, there exists parallels between these practices and practices in other spiritual traditions (e.g., prayer in Christianity and Islam) that can be drawn upon to increase the acceptability of these skills to clients of diverse backgrounds [31]. In one study, ethnicity, education, age, and marital status did not predict outcomes in self-harm behaviors, suicide attempts, remission, or dropouts following participation in DBT [32], suggesting that the treatment’s efficacy does not vary as a function of these demographic factors.

Considering the fact that elements of DBT make it a treatment easily adaptable for different cultural contexts, we were interested in examining the feasibility and effects of a pilot, 14-week DBT skills group on clinical outcomes in a sample of Muslim majority patients with BPD in Malaysia. Instead of introducing systematic adaptations to the content or format of the skills group a priori (with the exception of shortening the duration of the treatment), we intended to evaluate whether DBT skills, delivered largely as prescribed by the treatment manual [33], would hold promise in improving clinical outcomes among BPD patients in Malaysia. Given resource limitations (the lack of DBT-trained manpower resources in the country) and the fact that DBT is still a relatively new treatment modality in Malaysia, we developed and tested the acceptability and feasibility of a shortened, 14-week DBT skills group adapted from Soler and colleagues [19]. To date, no research has evaluated the effects of DBT or its components in the Malaysian context, though elements of DBT have reportedly been incorporated in an eclectic manner alongside other treatment approaches based on anecdotal case reports [34].

## Specific aims and hypotheses

The present study aimed to 1) evaluate the potential of a 14-week DBT skills group in improving clinical outcomes and well-being among patients diagnosed with BPD, and 2) assess the acceptability and feasibility of the skills group in a sample of Muslim-majority patients in Malaysia. We hypothesized that participation in the DBT skills group would be associated with significant reductions in self-harm behaviors, suicidal ideation, BPD symptoms, depression, anxiety, and difficulties with emotion regulation, as well as increases in subjective well-being and self-compassion from pre- to post-treatment. Feasibility of DBT skills training would be assessed through ability to recruit targeted sample size within the recruitment time frame (2 months) and rate of attrition from the skills group. Additionally, we examined qualitative feedback from patients in terms of satisfaction and perceived helpfulness of the DBT skills group (as an indicator of acceptability), skills that patients found more or less helpful, as well as suggestions for future implementation of the skills group in the Malaysian context.

## Methods

### Participants

A total of 20 psychiatric patients from a public hospital were recruited for the study. The study’s inclusion criteria included: 1) age between18–60, 2) meeting diagnostic criteria for BPD according to the Structured Clinical Interview for DSM Disorders- BPD Module (SCID-BPD), 3) able to read and understand English, 4) able to commit to attending 14 weeks of DBT skills group in the hospital’s outpatient clinic, and 5) having an attending clinician who agreed to assume primary responsibility for attending to elevated suicidal or self-harm risks throughout the study. The study’s exclusion criteria were: 1) experiencing persistent, active psychotic or manic symptoms, 2) having an organic brain disorder or intellectual disability, and 3) assessed to be experiencing imminent suicide risk (defined by expression of intent to kill oneself in the past 24 h) during intake assessment. Sample size calculation indicated that a sample size of 19 would be required to achieve .80 of power, assuming a moderate-to-large effect size (*d* = .60) and an alpha level of .05.

### Procedure

This study was approved by the research ethics committee of the National University of Malaysia. Eligible participants who consented to participate in the study were enrolled into a 14-week DBT skills training course. Patients were assigned to one of two concurrently running DBT skills groups, and each group consisted of 10 patients. All patients attended an intake session consisting of the SCID-BPD Module, a suicidality screener (Columbia Suicide Severity Rating Scale), and a battery of self-report questionnaires assessing self-harm behaviors, BPD symptoms, depressive symptoms, anxiety, stress, difficulties with emotion regulation, impulsivity, compassion, and well-being (see Measures section below). All measures were administered in English, and language proficiency was assessed by patients’ self-reported proficiency and observations by assessors during the intake session. Following the intake assessment, participants who did not meet eligibility criteria (e.g., not meeting full diagnostic criteria for BPD, demonstrating imminent suicide risk) were excluded from the study. The same battery of self-report questionnaires was administered to the patients again within 2 weeks after the end of the DBT skills groups. Following the intake assessment, one participant dropped out of the study as the participant relocated out of town. At the post-intervention assessment, participants also responded to several open-ended questions assessing their feedback (e.g., perceived helpfulness and level of satisfaction) regarding skills groups. These questions were administered using an anonymous online feedback form. Each participant was paid 50 Ringgit Malaysia (equivalent to approximately US$11) for attending an assessment session.

The sessions were delivered in English by several of the study investigators (three psychiatrists and two Masters-level therapists in training), who attended two in-person DBT training workshops, lasting for 5 days in total. The study therapists received weekly online supervision following a DBT consultation team format led by the first author, a doctorally-trained clinical psychologist who received her DBT training in the United States and Canada. The skills taught in the skills groups included four core modules of DBT, namely mindfulness (3 sessions), emotion regulation (4 sessions), distress tolerance (3 sessions), and interpersonal effectiveness (4 sessions). Each session was led by two co-leaders and lasted for 2 hours. Delivered based on protocols outlined in Linehan (2014), each session would begin with a brief mindfulness practice, followed by homework review and teaching of new skills as per standard DBT protocol (see Table 1 for an outline of the 14-week curriculum). The skills groups were provided at no financial cost to the research participants, and research participants continued to receive other forms of routine care available from the hospital (e.g., ad hoc or regular consultations with their psychiatrists) throughout their participation in the skills group.

**Table 1:** Outline of the 14-Week DBT Skills Group (Adapted from Soler et al., 2009)

Session	Module	Skills
1	Mindfulness	Orientation and Guidelines of Skills Training; Wise Mind
2		“What” Skills
3		“How” Skills
4	Distress Tolerance	TIPS Skills; Distracting; Self-Soothing; Improving the Moment
5		Reality Acceptance
6		Willingness; Mindfulness of Thoughts
7	Emotion Regulation	Understanding Emotions; Describing Emotions
8		Accumulating Positive Emotions
9		Building Mastery; Mindfulness of Current Emotions
10		Opposite Action
11	Interpersonal Effectiveness	Factors in the Way of Interpersonal Effectiveness; Clarifying Priorities in Interpersonal Situations
12		Objective Effectiveness: DEAR MAN
13		Relationship Effectiveness: DEAR GIVE; Self-respect Effectiveness: DEAR FAST
14		Evaluating Your Options (The Dime Game); Wrapping Up

Although the intervention protocol used in this study is based on the standard DBT skills training manual [35], we used specific, culturally relevant examples to explain the meaning and relevance of DBT skills in participants’ lives. For example, as the majority of the participants were Muslims, we highlighted the convergence of the concept of mindfulness and prayers in Islam. In particular, we used the term “kusyu” (reflecting the idea of wholehearted prayer) to clarify the idea of presence of mind, which is the opposite of the ‘automatic pilot’ mode [36].

### Measures

#### Demographic data form

A demographic information form was administered to assess participants’ gender, age, ethnicity, relationship status, past treatment history, and employment status.

#### Personality assessment inventory - borderline features scale (PAI-BOR) [37]

The PAI-BOR is a 24-item questionnaire developed to measure symptoms of BPD. The scale consists of four subscales: affective instability, identity problems, negative relationships, and self-harm. All items are rated on a 4-point Likert scale, ranging from 0 (“false, not true at all”) to 3 (“very true”). Examples of items include: “I’m careful about how I spend my money” and “Sometimes I feel terribly empty inside”. Higher scores indicate higher severity of BPD symptoms. The scale has been shown to have high internal consistency in nonclinical samples (Trull, 1995). In this study, the scale demonstrated good internal consistency (Cronbach’s alpha = .83).

#### Deliberate self-harm inventory (DSHI) [38]

The DSHI is a 17-item self-report questionnaire to assess the presence and frequency of various types of non-suicidal self-harm behaviors, ranging from cutting, burning, scratching, to hitting oneself, within the past 4 months. The inventory has been shown to have high internal consistency and adequate test-retest reliability in an undergraduate sample [38]. In this study, two variables were derived from the DSHI: overall frequency of self-harm behaviors (across all types endorsed) and number of types of self-harm behaviors.

#### Patient health Questionnaire-9 (PHQ-9) [39]

The PHQ-9 is a nine-item measure administered to assess current depressive symptoms experienced by participants in the last 2 weeks. Items are rated on a 4-point Likert-type scale, ranging from 0 (not at all) to 3 (nearly every day). Higher scores indicate greater severity of depressive symptoms. Additionally, item 9 (“Thoughts that you would be better off dead, or of hurting yourself”) was analyzed separately as a measure of suicidal ideation. The scale demonstrated good internal consistency (Cronbach’s alpha = .86) for the study’s sample.

#### Depression anxiety and stress scales (DASS-21) [40]

The DASS-21 is a well-validated measure to assess symptoms of depression, anxiety, and stress. In this study, the Anxiety and Stress subscales from the DASS-21 were administered to assess for current symptoms of anxiety and stress. Items are rated on a 4-point scale, from 0  ( did not apply to me at all) to 3  (applied to me very much, or most of the time). The DASS-21 and its subscales have demonstrated good to excellent internal consistencies in a Malaysian student sample [41]. In the present study, the internal consistencies for the anxiety subscale and the stress subscale were .86 and .83 respectively.

#### Difficulties with emotion regulation scale- short form (DERS-SF) [42]

The DERS-SF is a self-report multidimensional tool developed to assess emotional dysregulation. It consists of 18 items rated on a Likert-type scale, ranging from 1 (Almost Never) to 5 (Almost Always). Examples of the scale’s items include: “I pay attention to how I feel” and “When I’m upset, I become out of control.” The correlations between the original, 36-item DERS [38] and the DERS-SF ranged between 0.91 and 0.98 [42]. In the present study, the scale demonstrated high internal consistency (Cronbach’s α = .90).

#### Self-compassion scale (SCS) [43]

The SCS assesses the tendency to be kind toward oneself in times of difficulty or pain, to be nonjudgmental about one’s thoughts and feelings, and to see one’s experience as a part of the larger human experience. This 26-item questionnaire is composed of 6 subscales: self-kindness, self-judgment, common humanity, isolation, mindfulness, and over-identification. The items are rated on a 5-point Likert-type scale (1 = almost never; 5 = almost always), with higher scores indicating greater levels of self-compassion. In this study, the internal consistency of the SCS was excellent (Cronbach’s α = .91).

#### Personal well-being index (PWI) [44]

The PWI measures the degree of satisfaction experienced by individuals in multiple dimensions of their life. Participants rate their subjective satisfaction of their life as a whole and of eight specific domains (e.g., health, personal relationships, and spirituality) on a 10-point scale (0 = completely dissatisfied; 10 = completely satisfied). The scores on these items were added to form an overall score. In this sample, the internal consistency of the scale was good (Cronbach’s α = .84).

#### BPD module of the structured clinical interview for Axis II personality disorders (SCID-II) [45]

The SCID-II is a semi-structured interview for the diagnosis of personality disorders. During the intake assessment, the BPD module of the SCID-II was administered to assess the diagnosis of BPD among patients. The SCID-II demonstrated good test-retest and inter-rater reliability for the DSM-IV BPD section (Lobbestael et al., 2011; Maffei et al., 1997). To determine the interrater reliability of scores on the SCID-II in this study, two research assistants completed separate ratings for 10% of the participants. The intraclass correlation coefficient for two-way mixed model (using absolute agreement definition; ICCs) was .97 (excellent) for all individual SCID-II item scores.

#### Columbia suicide severity rating scale (C-SSRS) [46]

The C-SSRS (Screen Version-Recent) was administered as a screening tool to assess participants’ eligibility to participate in the study. It is an interviewer-rated scale to assess the presence of suicidal ideation in the past 1 month, in terms of severity (i.e., a specific plan or method) and intensity (i.e., frequency and duration). The C-SSRS screener is a 6-item abbreviated version of the C-SSRS full Columbia Protocol, which has well-established psychometric properties in terms of good convergent and divergent validity for suicidal ideation, and moderate to strong internal consistency for intensity of ideation subscale [46]. The cross-cultural validity of the C-SSRS full protocol is well-established [47] and has been used to assess suicide risk in Malaysian studies [48]. In this study, participants assessed to be at imminent suicidal risk (i.e., having intent and plan to engage in suicidal behavior within the past 24 h) were excluded from the study.

#### Post-DBT skills group feedback questions

At Time 2 assessment, all participants responded to several questions (administered online) assessing their feedback regarding the DBT skills group (see Supplementary Information for the list of questions). The first two questions assessed participants’ levels of satisfaction and perceived helpfulness of the skills group in addressing their problems, on a scale of 1 to 7 (1 = Not satisfied/helpful at all; 7 = Very satisfied/helpful). The remaining questions were open-ended questions assessing the impact of the skills group in their daily life. These questions included: 1) Please describe, in your own words, ways in which learning the DBT skills has impacted or benefitted you in your daily life? 2) What are the aspects of the DBT skills group you find the most helpful, or like the most? 3) What are the aspects of the DBT skills group you find less helpful? 4) Are there ways in which the skills group can be improved in the future? and 5) Is there anything else you would like to add?

### Data analytic plan

Data analyses were conducted using IBM SPSS. Boxplot analyses were run to check for outliers, and extreme outliers were replaced with the next highest score plus one unit above the score (Field, 2009). Descriptive statistics were reported for all variables. As one participant withdrew from the study immediately after Time 1 assessment, data analyses were carried out on the remaining participants who provided complete data (*n* = 19). A complete case analysis approach was adopted as there was no evidence of attrition bias (see section below). Given the small sample, nonparametric tests were used. Spearman’s rank order correlation analysis was conducted to examine the association among outcome variables at baseline. Wilcoxon signed-rank tests were run to examine changes in each outcome variable from pre- to post-intervention. To control for inflated Type I error due to multiple tested DVs, we applied Bonferroni correction (.05/10 tests) and set the significance threshold at α = .005.

Next, we carried out qualitative content analyses using guidelines established by Elo and Kyngäs (2008) to analyze participants’ open-ended feedback for the DBT skills group. We adopted an inductive content analysis approach, involving open coding, creation of categories, and abstraction to summarize participants’ feedback. Two coders independently coded the data prior to reviewing the codes to discuss any disagreements. Using procedures established by Hayes and Krippendorff [49], we calculated the inter-coder reliability based on 25% of all data units and derived a kappa value of .96, which reflects excellent reliability. The analyses also quantified participants’ feedback based on the frequencies with which each code was endorsed by the participants [50]. Following recommendations by MacDougall and Baum [51], the first author served as the devil’s advocate to challenge assumptions made and present alternative interpretations or coding of the data.

## Results

### Sample characteristics

We successfully recruited 20 patients within the stipulated time frame of recruitment for the study (2 months; between August and October of 2019).^1^ The mean age of the participants was 27.25 years (range = 19–45; *SD* = 7.67). Of the twenty participants, most were female (95%; *n* = 19). Close to half were single (45%; *n* = 9), whereas the remaining were married or in an intimate relationship (40%; *n* = 8), or divorced or separated (15%; *n* = 3). The majority of the participants were Malays (80%; *n* = 16), followed by Chinese (10%, *n* = 2), Indians (5%, *n* = 1), and others (5%; *n* = 1). With regard to education, 35 % (*n* = 7) completed a minimum of a Bachelor’s degree, whereas the remaining (65%; *n* = 13) did not complete, or have not completed university. Sixty-percent (*n* = 12) of the overall sample were university students. Thirty percent (*n* = 7) of the sample were working part time or full time, with the remaining not employed (70%; *n* = 14). Further, 70 % (*n* = 14) of the participants had previously been hospitalized for suicidal or self-harm behaviors. All participants were on psychotropic medications at the time of enrolment. Based on information acquired from medical records, four participants (20%) had a comorbid diagnosis of bipolar I or II disorder, and another four (20%) had a comorbid diagnosis of major depressive disorder. One participant was additionally diagnosed with posttraumatic stress disorder and social anxiety disorder, whereas another participant had a comorbid diagnosis of attention-deficit hyperactivity disorder. Mann-Whitney U tests indicated no significant differences between participants who completed the study versus the participant who dropped out on any of the outcome variables at baseline, *p*s > .30, indicating no differential attrition bias.

### Preliminary analyses

Table 2 presents Spearman’s rank order correlations among all outcome variables at baseline. As expected, there were significant associations among several variables. For example, severity of BPD symptoms was positively and significantly associated with depressive symptoms, anxiety symptoms, stress symptoms, impulsivity, difficulties with emotion regulation, and negatively associated with self-compassion and personal well-being. Frequency of deliberate self-harm behaviors was significantly and positively associated with number of types of self-harm behaviors, and marginally associated with suicidal ideation and difficulties with emotion regulation.

**Table 2:** Spearman’s Rank Order Correlations among Outcome Variables at Baseline

	1	2	3	4	5	6	7	8	9	10
1. PAI-BOR Total	–									
2. DSHI Frequency	.27	–								
3. DSHI Types	.24	.70**	–							
4. PHQ-9 Tota	.43^t^	.41^t^	.26	–						
5. PHQ-9 Suicidal Ideation	.13	.24	.30	.61**	–					
6. DASS-Anxiety	.47*	.28	.32	.20	.22	–				
7. DASS-Stress	.60**	.10	.20	.12	.20	.78**	–			
8. DERS Total	.72**	.44^t^	.25	.46*	.15	.41^t^	.46*	–		
9. SCS Total	−.56*	.05	−.05	.06	.12	−.29	-.44^t^	−.49*	–	
10. PWI Total	−.60*	−.05	−.15	−.26	−.02	−.34	−.29	−.49**	.60**	–

*Notes*: *PAI-BOR* Personality Assessment Inventory – Borderline Subscale, *DSHI* Deliberate Self-Harm Inventory, *PHQ-9* Patient Health Questionnaire- 9, *DASS* Depression, Anxiety, and Stress Scales, *DERS* Difficulties with Emotion Regulation Scale, *SCS* Self-Compassion Scale, *PWI* Personal Well Being Index. ^t^ *p* < .10; ^*^p < .05; ^**^p < .01

Of nineteen participants who completed the study, seventeen (89.47%) completed a minimum of ten (approximately 70%) out of fourteen sessions. The remaining participants completed eight and nine sessions respectively. None of the patients dropped out from the skills group (defined as missing three consecutive sessions in the row).

### Pre- to post-intervention change on all outcome variables

Results from Wilcoxon signed-rank tests indicated that there were significant decreases in borderline personality disorder symptoms, depressive symptoms, stress, and difficulties with emotion regulation, as well as increases in self-compassion and personal well-being from Time 1 to Time 2 (see Table 3), all *p*s < .005. There was a trend for reductions in frequency and types of non-suicidal self-harm behaviors, suicidal ideation, and anxiety symptoms from pre- to post-intervention, *p*s ranging from .019 to .088. Effect sizes were largely in the moderate-to-large range.

**Table 3:** Descriptive and Test Statistics for Changes in All Outcome Variables from Time 1 to Time 2

Variable	Time 1 M (SD)	Time 2 M (SD)	*Z*	*p*	Effect Size (*r)*
PAI-BOR Total	52.59 (9.55)	47.00 (9.29)	−2.84	.004	.65
DSHI Frequency	13.50 (15.46)	5.82 (6.03)	−2.10	.036	.48
DSHI Types	2.75 (2.02)	1.95 (1.68)	−2.20	.028	.50
PHQ-9 Total	22.05 (5.32)	16.21 (7.36)	−3.22	.001	.74
PHQ-9 Suicidal Ideation	2.50 (.89)	1.63 (1.26)	−2.35	.019	.54
DASS-Anxiety	14.35 (5.46)	11.63 (6.16)	−1.71	.088	.39
DASS-Stress	16.30 (4.05)	11.11 (5.76)	−3.42	.001	.78
DERS Total	66.75 (11.20)	56.79 (15.16)	−2.90	.004	.67
SCS Total	56.75 (15.75)	69.53 (15.42)	3.35	.001	.77
PWI Total	24.00 (14.16)	36.00 (14.26)	3.12	.002	.72

*Notes*: *PAI-BOR* Personality Assessment Inventory – Borderline Subscale, *DSHI* Deliberate Self-Harm Inventory, *PHQ-9* Patient Health Questionnaire- 9, *DASS* Depression, Anxiety, and Stress Scales, *DERS* Difficulties with Emotion Regulation Scale, *SCS* Self-Compassion Scale, *PWI* Personal Well Being Index

### Summary of participants’ feedback for the DBT skills group

On average, participants’ level of satisfaction toward the skills group was 5.47 (*SD* = 1.54), on a 1 to 7 scale. The mean score of perceived helpfulness of the skills group was 5.58 (*SD* = 1.50). With regard to the perceived impact of the skills group, eighteen participants (95%) expressed that the DBT skills group had a positive impact on them. Six participants (31.6%) reported that the skills group has helped them in improving regulation of their emotions. Three participants (15.8%) highlighted that DBT skills group had increased their self-awareness in general. Several participants (*n* = 6; 31.6%) reported that the skills helped increase their confidence or sense of efficacy in handling problems in daily life. Two (10.5%) participants highlighted that the skills group helped increase their awareness of BPD symptoms or acceptance of the diagnosis of BPD. Three (15.8%) participants expressed appreciation towards the structured delivery and teaching of DBT skills (e.g., provision of a handbook and weekly homework). Other aspects that participants described as helpful included a positive and non-judgmental group environment, professional guidance and teaching from the facilitators, and peer support from other group members.

Of all the DBT skills modules, distress tolerance and mindfulness were frequently rated as skills that participants found beneficial. Eight participants (42.1%) indicated that distress tolerance skills helped them with enduring their pain while coping and surviving a crisis. Seven participants (36.8%) noted that mindfulness practices helped them to be more grounded in the present moment. Five participants (26.3%) reported that Interpersonal Effectiveness skills helped them in building and maintaining healthy interpersonal relationships, while three participants (15.8%) specified that emotion regulation skills were helpful.

Meanwhile, participants described challenges in learning some of the skills, including mindfulness, distress tolerance (i.e., radical acceptance), and interpersonal effectiveness (e.g., DEAR MAN). Six participants (31.6%) reported that the duration of the skills group was too compact for them to learn all the DBT skills thoroughly. There was therefore an expressed desire for longer sessions, or for the skills modules to be repeated to facilitate more in-depth learning of DBT skills. Five participants (26.3%) highlighted the need for greater availability of DBT services in Malaysia, including a suggestion for DBT to be a part of government-sponsored healthcare.

## Discussion

This study examined the feasibility, acceptability, and outcomes of a 14-week DBT skills group in a sample of 20 Malaysian patients diagnosed with BPD. Results showed that there were significant decreases in the BPD symptoms, depressive symptoms, stress, and difficulties with emotion regulation, as well as increases in well-being and self-compassion from pre- to post-intervention, with effect sizes mostly in the moderate-to-large range. There was a trend for reductions in number and types of nonsuicidal self-harm behaviors, suicidal ideation, and anxiety symptoms. Overall, participants reported a high level of satisfaction with the skills group, with a majority indicating that the skills group had a positive impact on them. The skills group also demonstrated feasibility, as reflected by the study’s success in recruiting the target sample within the recruitment time frame and a zero dropout rate. Further, the skills group leaders were able to administer the skills groups under regular supervision by a trained DBT therapist.

The present study demonstrated promising clinical outcomes following implementation of a shortened (i.e., 14-week) DBT skills group among diagnosed BPD patients. The findings suggest that DBT skills could yield positive outcomes, including reductions in several psychopathological symptoms such as BPD symptoms, depressive symptoms, and stress, even if taught within a short time window. Reductions in difficulties with emotion regulation corresponds with the primary aim of the treatment, which targets emotion dysregulation as a core feature of BPD [14, 52]. Specifically, a number of DBT skills such as mindfulness “how” and “what” skills and emotion regulation skills could enable patients to learn to be more aware of their thoughts and emotions nonjudgmentally, and to develop more adaptive strategies to regulate their emotions.

Importantly, the skills group was also associated with significant increases in self-compassion and personal well-being, suggesting that the intervention might have an impact above and beyond reducing psychopathological symptoms. The finding suggests that selected DBT skills learned by patients (e.g., dropping self-judgments, increasing engagement in pleasant activities, and reducing vulnerability to negative emotions) might enable them to relate to themselves and more compassionately, and thereby experience a greater sense of well-being. There was a nonsignificant trend in the reduction of non-suicidal self-harm behaviors, suicidal ideation, and anxiety from pre- to post-intervention, suggesting that a longer duration of skills training may be required for participants to learn adequate skills to decrease self-harm behaviors and suicidality, and to modulate anxiety effectively. Incorporation of individual therapy consistent with the DBT model (e.g., regular use of commitment strategies and individualized coaching from therapists) may also yield better effects with regards to reducing self-harm behaviors and suicidality. Specific adaptations (e.g., formal incorporation of exposure protocol) may also be required for DBT skills training to exert a larger effect on anxiety-specific outcomes, as demonstrated by a study by Harned and colleagues [35].

Notably, nearly 90% of participants completed 70% (ten out of 14 sessions) of the DBT skills group. With the exception of one patient who relocated to a different city prior to the initiation of the skills group, no participant dropped out of the study or the DBT skills group (as defined by missing three consecutive sessions in a row) upon its commencement. The zero dropout rate is highly encouraging, especially given that past research on DBT skills groups have demonstrated higher dropout rates (e.g., 32% for Neacsiu and colleagues [22]). One reason underlying the high completion rate could be due to nature of the current sample, in which a number of patients were highly motivated to receive DBT given that this was the first ever DBT skills group implemented for patients in Malaysia. Further, the treatment was offered at no cost to the patients. Notably as well, the treating clinicians received ongoing supervision support throughout the implementation of skills group, and ad hoc efforts were made to reach out to patients and their individual primary treating clinicians (with the goal of re-engaging patients in treatment) if patients were found to be at risk of dropping out of the skills group. In other words, the DBT skills group examined in this study might be better considered as a “DBT skills+ group” given the additional efforts put forth in engaging patients in the study, despite the absence of formal individual DBT sessions or phone coaching that accompany a standard treatment. It is worth noting that the present sample represents a relatively high risk sample, given that we did not exclude patients who had engaged in recent suicidal behaviors, and 70% of the sample had a history of hospitalizations due to suicidal or self-harm behaviors.

A large majority of participants (95%) reported that DBT skills group has made a positive impact on them, with distress tolerance and mindfulness cited most frequently as skills that many patients found beneficial. In line with findings acquired from quantitative analyses, some participants reported improved awareness of, as well as ability to regulate emotions following attendance of the skills group. Some participants reported that the DBT skills group sessions were too compact and suggested for longer sessions. Therefore, even though a shortened adaptation (14 weeks) of DBT skills training demonstrated positive outcomes, participants indicated a preference for more or longer sessions. The feedback suggests that offering booster sessions or repeating the skills modules might lead to better and more sustained clinical outcomes, as they offer participants an opportunity to consolidate their learning and practice of DBT skills. Also, while carrying out a comprehensive DBT program may not be feasible given a lack of trained DBT therapists in the country, institutions interested in offering DBT skills training could consider engaging non-DBT trained therapists (as what was done in this study) to serve as case managers or primary attending clinicians for BPD patients as they attend the skills group. This treatment approach has received some empirical support in a study involving dismantling of components of DBT [20]. It was encouraging that patients in this study observed the need for greater availability of DBT services in Malaysia, and this may encourage further efforts within the hospital and the Malaysian government to devote resources to support dissemination of evidence-based interventions.

Anecdotally, the skills group leaders observed challenges described by patients in learning selected DBT skills. For example, many patients were living with their family and found it challenging to practice DEAR MAN in their home environment due to existing family dynamics, such as an expectation for children to be deferential towards their parents. Further, several participants expressed concern regarding stigma associated with being diagnosed with BPD. For example, one patient resorted to hiding under a table when she became concerned whether someone she knew might be outside of the clinic room when she was participating in the skills group. While BPD is a condition that is also often stigmatized in other contexts [53], there may be added value to address the experience of stigmatization in the Malaysian context. Future research should explore whether other more systematic cultural adaptations (e.g., navigating the dialectic between respecting parents’ wishes versus expressing one’s needs when they are in conflict with parents’ expectations) of the protocol would increase participants’ engagement in DBT skills training and the treatment’s efficacy.

The present study is the first study demonstrating the feasibility and potential benefits of a DBT skills group in Malaysia. Despite preliminary in nature, the findings lend support to the cross-cultural generalizability of the effects of DBT skills training, in a context in which the majority of patients are Malay Muslims, which represents the largest ethnic group in Malaysia. The findings also indicate that it is feasible to conduct a DBT skills group in a resource-scarce setting. The translation of such encouraging findings beyond research settings into sustainable models of routine clinical practice would require judicious resource allocation and investments in terms of manpower, training and ongoing supervision of treatment providers. Future cost-benefit analysis research would further build the evidence-base for successful implementation of DBT skills group in routine care. Use of a semi-structured interview increases the reliability of the diagnosis of BPD in this sample. Further, provision of the skills group within the setup of routine mental health services in a public hospital setting enhances the external validity of the findings.

The findings should be interpreted in light of several limitations. Importantly, a small sample size and the lack of a control group preclude causal conclusions with regard to the efficacy of DBT skills group. As patients in the study were receiving other concurrent treatments (e.g., medications and varying number and frequency of individual consultation sessions with their psychiatrists) while in the study, the effects observed cannot be fully attributable to the DBT skills group. Future research would also need to rule out the effects of other confounding factors, such as expectancy effect and group support, which may influence treatment outcomes. Further, use of self-report measures to assess outcomes is susceptible to biases such as social desirability and demand characteristics. Therefore, the findings should be interpreted as preliminary, and future research is needed to evaluate the efficacy of DBT with a larger sample and using a more rigorous design, such as a randomized controlled trial and inclusion of objective measures (e.g., assessment of diagnoses and functioning by blind interviewers) to assess clinical outcomes. Inclusion of follow-up assessments would also enable evaluation of longer-term outcomes following from attendance of the skills group. Due to a lack of sufficient research manpower, post-intervention qualitative feedback assessments were carried out by two of the study therapists, which might lead to expectancy bias among the participants. However, the bias could be mitigated to some extent through the use of a self-report form administered online (as opposed to a verbal interview) to collect anonymized feedback. Lastly, the findings have limited generalizability given the nature of the sample as a self-selected sample of majority Muslim participants from a large urban city who were proficient in English. In contrast to the sample of this study, approximately 50% of Malaysians are literate in English [54]. Meanwhile, the National language of Malaysia is Malay, with other languages such as Chinese and Tamil being routinely spoken by ethnic minority groups of Chinese and Indians respectively. Future research could translate DBT’s treatment materials into other languages commonly spoken in Malaysia (and other parts of Asia), such as Malay, Mandarin Chinese, and Tamil, to enable a broader dissemination and evaluation of the treatment in diverse linguistic and cultural contexts. Lastly, even though a number of outcomes were associated with moderate-to-large effect sizes, participants’ mean post-intervention scores on several outcomes (e.g., PAI-BOR and PHQ-9) remained in the clinical range, indicating limited clinical significance of the findings. Future work should examine whether more intensive or longer treatments may yield better outcomes that translate into improved functioning.

To summarize, this study demonstrated that a 14-week DBT skills training group was feasible and acceptable in a Malaysian outpatient mental health facility, in the presence of strong institutional support and a highly motivated treating team. The results were encouraging, as reflected by significant reductions in selected psychological symptoms and difficulties with emotion regulation, as well as improvements in self-compassion and subjective well-being from pre- to post-intervention. Future research should validate these findings using a larger sample and a controlled group design, as well as examine the generalizability of the effects among patients from other cultural and linguistic backgrounds in Malaysia. It would also be a worthwhile effort to examine whether systematic, cultural adaptations of the treatment may improve the efficacy of DBT in this context [55, 56]. While full mode DBT, when available, will likely provide even greater benefits to patients with BPD, the findings suggest that DBT skills training groups are a feasible and promising first step to address the clinical needs of complex patients in a low resource public hospital setting in Malaysia.

## Supplementary Information

**Additional file 1.**

## Data Availability

The dataset supporting the conclusions of this article is available in the Open Science Framework, https://osf.io/e69vt/.

## Footnotes

1 A total of 45 patients were invited for the study, with six declining to participate (reflecting an initial refusal rate of 13%). The remaining 39 participants attended the intake interview, out of whom 27 were found to meet study criteria. Seven eligible participants chose not to enrol in the study, leaving the final sample size to be 20.
