# Supplementary material for: Implementation of brief dialectical behavior therapy skills training among borderline personality disorder patients in Malaysia: feasibility, acceptability, and preliminary outcomes
Source: BMC Psychiatry. 2021 Oct 4;21:486. doi: 10.1186/s12888-021-03500-y (PMC8489091; doi:10.1186/s12888-021-03500-y)
Supplement: Supplementary file 1 — Additional file 1. [file 12888_2021_3500_MOESM1_ESM.docx]

Implementation of Brief Dialectical Behavior Therapy Skills Training among Borderline Personality Disorder Patients in Malaysia: Feasibility, Acceptability, and Preliminary Outcomes

Shian-Ling Keng^1^, Hajar Binti Mohd Salleh Sahimi^2^, Lai Fong Chan^2^, Luke Woon^2^, Choon Leng Eu^2^, Su Hua Sim^3^, & Man Kuan Wong^3^

1. Division of Social Scinece, Yale-NUS College, Singapore
2. Department of Psychiatry, Hospital Canselor Tuanku Muhriz, National University of Malaysia (UKM), Malaysia
3. Department of Psychology, HELP University, Malaysia

**Supplementary Information**

Post DBT Skills Group Feedback Questions

1. On a scale of 1 to 7, how satisfied were you with the DBT skills group? (1 = Not satisfied at all; 7 = Very satisfied).
2. On a scale of 1 to 7, how helpful did you find the DBT skills group? (1 = Not helpful at all; 7 = Very helpful).
3. Please describe, in your own words, ways in which learning the DBT skills has impacted or benefitted you in your daily life?
4. What are the aspects of the DBT skills group you find the most helpful, or like the most?
5. What are the aspects of the DBT skills group you find less helpful?
6. Are there ways in which the skills group can be improved in the future?
7. Is there anything else you would like to add?
